# Study on the failure mechanism of high-temperature granite under two cooling modes

**DOI:** 10.1038/s41598-024-66073-2

**Published:** 2024-07-07

**Authors:** Chun Li, Gan Feng, Xinran Zhang, Chunwang Zhang, Yaoqing Hu, Tao Meng

**Affiliations:** 1 School of Mining Engineering, Taiyuan University of Technology, Taiyuan, 030024 Shanxi China; 2 State Key Laboratory of Hydraulics and Mountain River Engineering, College of Water Resource & Hydropower, Sichuan University, Chengdu, 610065 China; 3 Heilongjiang Geology and Mineral Resources Investment Group Co., Ltd., Harbin, 150090 China; 4 Nenjiang Kunyuan Mining Co., Ltd., Heihe, 161400 China; 5 Center of Shanxi Engineering Research for Coal Mine Intelligent Equipment, Taiyuan University of Technology, Taiyuan, 030024 China; 6 Key Laboratory of In-situ Property-improving Mining of Ministry of Education, Taiyuan University of Technology, Taiyuan, 030024 Shanxi China; 7 Oil and Gas Storage and Transportation Engineering Laboratory, College of Chemistry and Biological Engineering, Taiyuan University of Science and Technology, Taiyuan, 030024 Shanxi China

**Keywords:** Energy science and technology, Engineering

## Abstract

In the geothermal development of hot dry rock (HDR), both the drilling of the wellbore and the heat exchange of the heat reservoir involve the effects of different cold and hot conditions on the high-temperature rock mass. The testing machine for rock mechanics was used to conduct a uniaxial compression test and carry out micro testing on the treated samples; furthermore, with the help of scanning electron microscopy the fracture mechanism of granite subjected to different temperatures and cooling methods was studied. The results show: (1) With the gradual increase in temperature, the compressive strength of granite under the two cooling methods gradually decreases. (2) The failure modes of the samples under the two cooling methods are mainly shear failure of the "Y" type. The degree of damage of the sample under water cooling is significantly greater than that under natural cooling. Electron micrographs could confirm these results. (3) It can be obtained by testing the mineral composition and element changes of granite at different temperatures. When the temperature reaches 600℃, its change is more pronounced. The results of this study can provide a theoretical reference for the failure of the wellbore and the degree of fracture of the thermal reservoir rock mass during geothermal development.

## Introduction

The main components of hot dry rock (HDR) geothermal mining are the drilling of wellbores into HDR and the construction of an artificial thermal reservoir^1,2^. During the wellbore drilling process, the local temperature of the rock mass drops sharply when the high-temperature rock body encounters water, leading to thermal cracking. On the one hand, this is beneficial for breaking the rock in the wellbore and thus improving the drilling efficiency. On the other hand, this causes the mechanical properties of the surrounding rock to change, which may lead to deformation and damage in the walls of the wellbore, the collapse of the wellbore, the occurrence of stuck drills, and even the scrapping of the wellbore. In geothermal mining in HDR, the artificial reservoir layer also experiences alternating cold and hot conditions^3^. Due to the thermal expansion and contraction of the rock during this process^4^, HDR undergoes cold cracking. New cracks are generated, and primary cracks expand or penetrate. The physical and mechanical properties of the pore structure and microstructure of HDR are changed, which leads to changes in the permeability characteristics of the artificial thermal reservoir, ultimately affecting its heat-exchange efficiency.

In recent years, the variation in the mechanical properties of granite has been analyzed^5,6^. For example^7,8^, they carried out an experimental study on granite after exposure to high temperature and under different confining pressure treatments. They reported that the peak stress and elastic modulus of the sample decreased with increasing the temperature it had been exposed to. Xiao-li^9^ studied the effect of temperature on the mechanical characteristics and behaviors of granite. The author conducted several experiments, including scanning electron microscopy (SEM), X-ray diffraction (XRD) and acoustic emission (AE), and discussed the micromechanism of the brittle-plastic transition of granite under high temperature. Secondly, some researchers^10–13^ studied the effects of different cooling methods on the mechanical properties of granite. The results show that heating and cooling have an important influence on the macroscopic physical and mechanical properties as well as the microscopic characteristics of rocks. Fan^14^ and Xiao-li^15^ studied the microscopic damage mechanism. They showed that the amount of thermal damage inside the rock increases as the temperature increases. Yang^16^ concluded that microscopic thermal cracking and porosity changes in granite are the main causes for the change in its strength and deformation characteristics as the temperature increases. Zuo^17^ used high-temperature SEM to test the fracture behavior of Beishan granite after heat treatment at 125 ~ 600 °C. Microcracks and an uneven thermal expansion deformation of the sample surface were observed at different temperatures alongside with long and deep cracks at grain boundaries. Additionally, the initial direction of the microcracks and the propagation path of the main cracks is affected by the shape of the mineral particles and the distribution of the thermal cracks.

The above works conducted experiments on granite under natural cooling conditions. However, cold water tests are generally used for heat exchange in enhanced geothermal systems. Therefore, in this work, the macroscopic physical and mechanical properties and microscopic failure characteristics of granite after water cooling from different temperatures are systematically investigated through uniaxial compression testing and fracture feature analysis. Identical experimental analyses are also conducted on such granites after natural cooling to allow for experimental comparison. The results of this study can provide values of technical parameters that are useful for the stable mining of enhanced geothermal systems^18–20^.

## Experimental work

**Figure 1:**
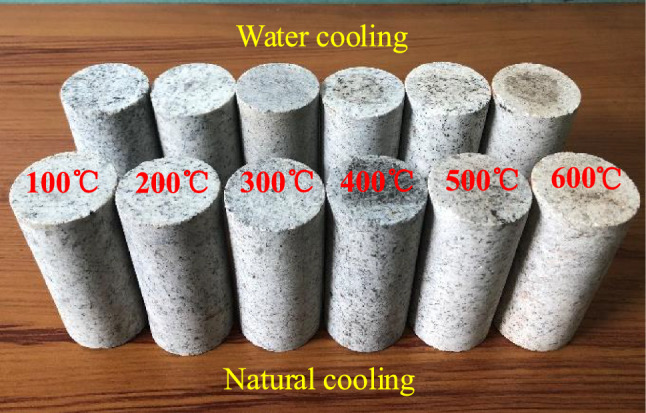
Granite specimens.

The test specimens had dimensions of *Φ* = 50 × 100 mm, and they were prepared according to the standards of precision recommended by the International Society for Rock Mechanics (ISRM). The specimens are shown in Fig. 1. The test specimens were divided into seven groups to be heated to different temperatures. The heating temperatures adopted in this study were: room temperature (20 ℃), 100, 200, 300, 400, 500, and 600 °C. Each group contained four specimens, yielding a total of 52 specimens. The specimens were heated to the different target temperatures at a constant rate of 4 °C/min, and the target temperature was then maintained for 8 h to ensure that the samples could be fully heated. A group of heated samples was directly taken out for water cooling and cooled to room temperature. The other group was placed in a furnace and cooled to room temperature.

**Figure 2:**
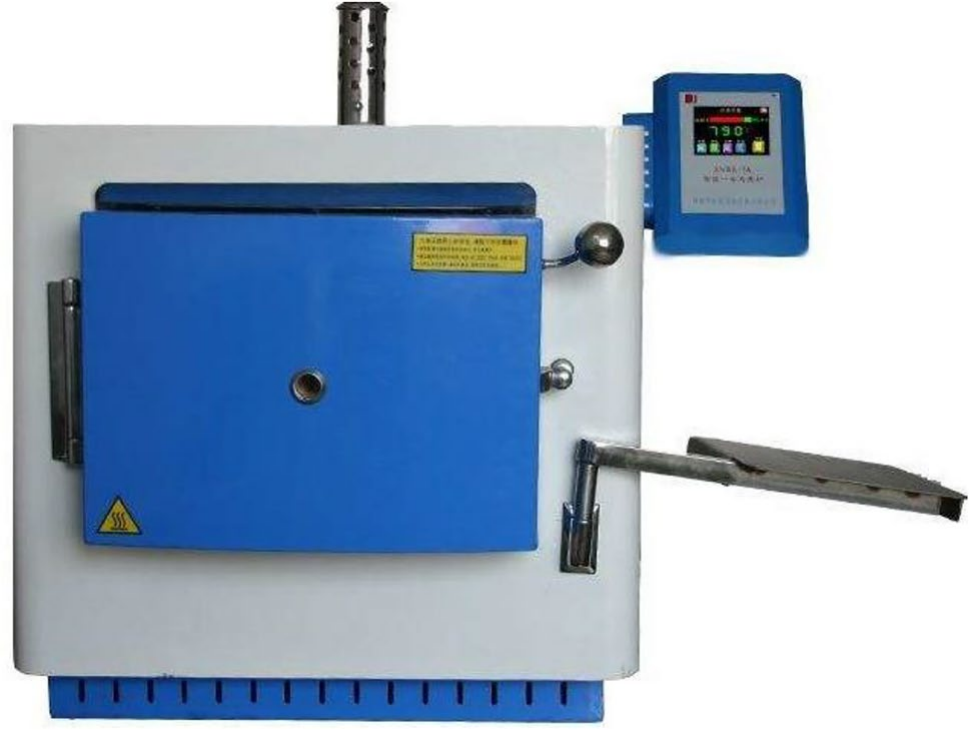
Heating equipment.

A full uniaxial stress–strain test was carried out on each test specimen using a rock testing machine. The loading mode was set to be displacement control with a loading rate of 0.02 mm/min. The test results were automatically recorded by the computer^21^. The cross-sectional morphology of the specimen was analyzed via SEM (model JSM-7610F). The specimens were heated up to 100 ~ 600 ℃ at a heating rate of 4℃/ min. The heating device was an XRMF-9X intelligent integrated muffle furnace, shown in Fig. 2. The main parameters were as follows: the heating temperature was from room temperature to1200 ℃, the resolution was is 1 ℃, and the temperature control accuracy was ± 1 ℃.

## Results

### Stress–strain curve

**Figure 3:**
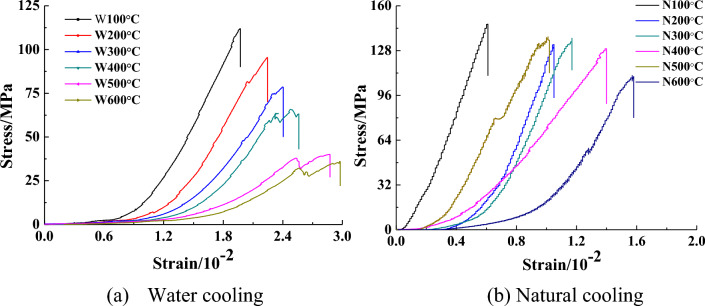
Stress–strain curves of granite under different temperatures and cooling modes^22^. (**a**) Water cooling, (**b**) Natural cooling.

It can be seen from Fig. 3a that the uniaxial compressive strength of the specimen under water cooling decreases with increasing temperature, while the peak strain increases with increasing temperature. The compaction phase of the specimen is also higher than that of the sample after natural cooling. In the elastic phase, it can be seen that the slope of the sample gradually decreases. When the temperature reaches 500 °C, it can be seen that the sample gradually changes from being brittle to being ductile.

It can be seen from Fig. 3b that the uniaxial compressive strength of the sample under natural cooling exhibits a downward trend with increasing temperature; however, this decrease is small, and the peak strain increases with temperature. An increasing trend can be observed, and the length of the compaction phase in the stress–strain curve of this sample is shorter than that of the sample under water cooling.

By comparing panels (a) and (b) in Fig. 3, it can be concluded that the strength of the sample under water cooling is significantly lower than that of the sample under natural cooling, and the peak strain is higher than that of the sample under natural cooling. Furthermore, the slope of the strain–stress curve for the sample under water cooling is less steep than that of the sample under natural cooling. It is especially noteworthy that the proportion of the compaction stage is relatively high. This may be due to the fact that the sample is affected by thermal expansion and contraction during heating and cooling, which causes cracks between the internal particles; thus, the time required for the pore cracks to be gradually compacted during the uniaxial compression process is longer.

### Failure mode

**Figure 4:**
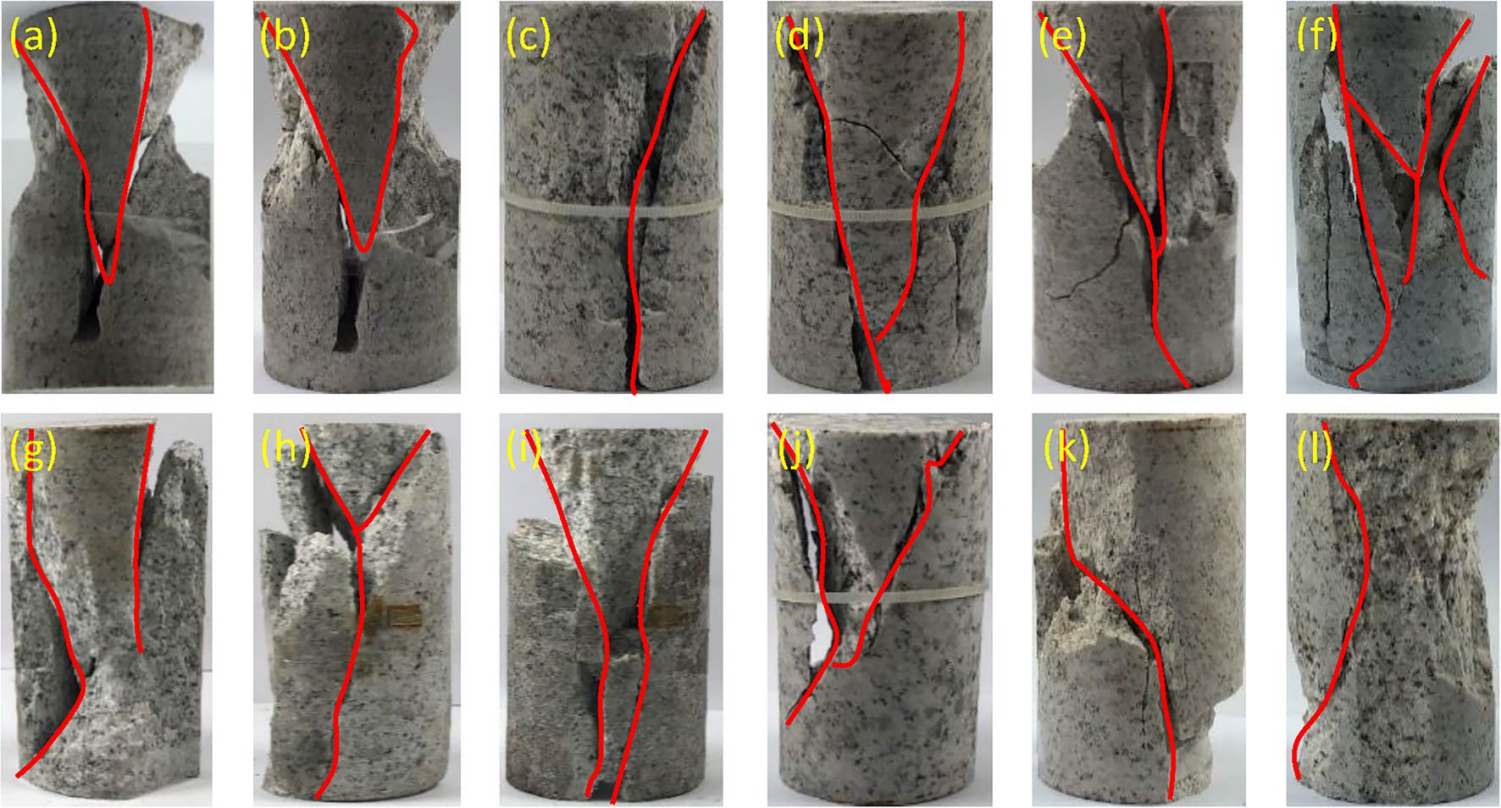
Failure modes of granite specimens with increasing temperature: (**a**–**f**) water cooling from 100 ~ 600 ℃ and (**g**–**l**) natural cooling from 100 ~ 600 ℃.

It can be seen from Fig. 4 that there are noticeable oblique shear planes and longitudinal penetrating planes (see panels (a, c ~ e) in the failure mode of granite after water cooling, and most specimens with conical shape are destroyed. More than 90% of the sample exhibit a conical destruction at the upper end and a splitting failure at the lower end. These are clearly Y-shaped and V-shaped, respectively. The upper part has a conical shape due to the end effect, while the longitudinal end of the crack is due to the lateral tensile failure caused by the pressure; the failure mode of granite after natural cooling is mainly caused by splitting damage. With the increase in temperature, the damage becomes increasingly more complex. When the temperature is 100 °C, the fragmentation of the specimen does not give rise to a macroscopic fracture surface. When the temperature is 200 °C, the macroscopic fracture surface has a conical shape. The fracture then becomes longitudinally penetrating and cracking when the temperature is 300 °C. At 400 °C the specimen exhibits a Y-type failure, while, when the temperature is 500 ~ 600 °C, the failure mode is more complex. When damage occurs, more particulate debris is produced^23^.

In general, the damage caused after cooling with water is mostly shear failure. The damage caused after natural cooling is mostly splitting and tensile failure. Although both are brittle modes of failure, the brittleness after cooling with water is smaller than that after natural cooling. Thus, the temperature has a significant effect on the failure mode.

### Analysis of the granite fracture images with SEM

SEM and polarized light microscopy were used to observe the fracture morphology of granite heated to different temperatures and subjected to different cooling modes.

**Figure 5:**
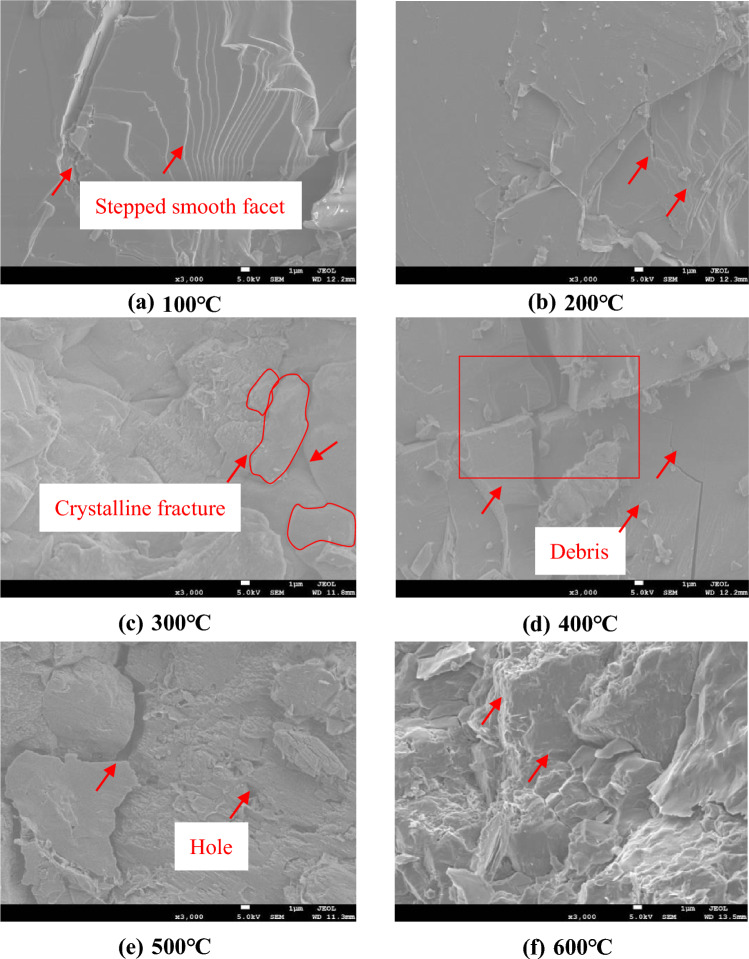
Changes in the microstructure of granite under water cooling (magnification: 3000). (**a**) 100 ℃, (**b**) 200 ℃, (**c**) 300 ℃, (**d**) 400 ℃, (**e**) 500 ℃, (**f**) 600 ℃.

Figure 5 shows SEM images of the fracture surfaces of granite specimens that were water-cooled after being heated to different temperatures. When the heating temperature is 100 °C (Fig. 5a), the microscopic fracture morphology of the sample is stepped, the crystal surfaces are smooth and flat, the structure is complete, and only small microscopic cracks are visible. When the heating temperature is 200 °C (Fig. 5b), the fracture morphology is smooth and flat, and one narrow unconnected transgranular microcrack can be noticed. When the heating temperature rises to 300 °C (Fig. 5c), narrow cracks and transgranular microcracks develop at grain boundaries, and micropores and a triangular micropit can be observed. When the heating temperature is 400 °C (Fig. 5d), noticeable large cracks appear on the fracture surface, which are interpenetrated. The fracture morphology is cleavage-stepped, and the main cracks meet to form a cross. Additionally, debris can be observed on the fracture surface of the sample. When the heating temperature is 500 °C (Fig. 5e), part of the crystal is destroyed, split by a large transgranular crack that communicates with an intergranular crack. The presence of fine transgranular cracks and distributed microcavities indicates that a slight plastic deformation of the fracture surface has occurred. This may be due to the fact that brightness is related to conductivity. When the heating temperature rises to 600 °C (Fig. 5f), the SEM image of the sample is brighter than at lower temperatures, indicating that rock heated to this temperature has high conductivity. Thus, heating has a pronounced effect. At this high temperature, the crystal surface is rough and broken, and the width of the crack along the grain boundary is greater than that at lower temperatures. After the thermal stress inside the sample reaches a certain level, grain boundaries and transgranular cracks will penetrate each other to form a fracture network, causing severe damage.

**Figure 6:**
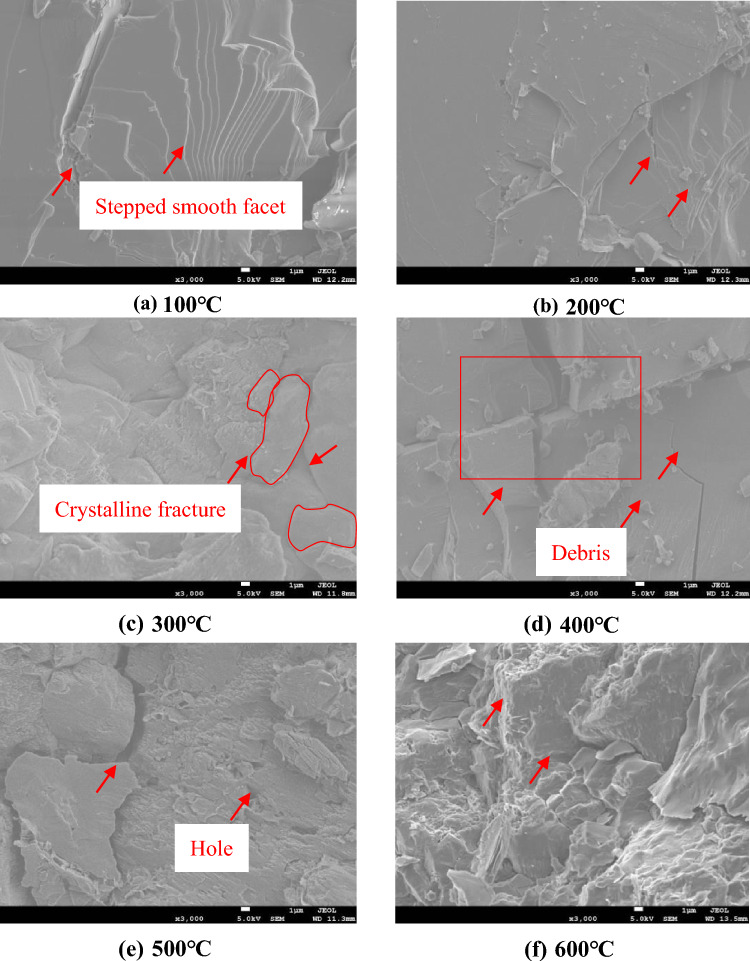
Changes in the microstructure of granite under natural cooling. (**a**) 100 ℃, (**b**) 200 ℃, (**c**) 300 ℃, (**d**) 400 ℃, (**e**) 500 ℃, (**f**) 600 ℃.

Figure 6 shows SEM images of the fracture surfaces of granite specimens cooled naturally after being exposed to different temperatures. Overall, the surface topography in Fig. 6a, b is smooth and flat with some minor cracks. From Fig. 6c, it can be seen that, when the heating temperature reaches 300 °C, the surface of the granite sample becomes deformed along the grain boundary, and microruptures like those seen at 100 and 200 °C have expanded and transformed to follow the grain boundary. Fig. 6d, e show that the surface is greatly damaged upon heating to 400 and 500 °C, becoming increasingly roughened and uneven, and that a small amount of debris is generated. Particles also start to peel off from the specimen. Figure 6f shows that, when the heating temperature reaches 600 °C, the surface of the sample is broken, pulverizing damage occurs, and more debris appears on the surface. These observations explain why the mechanical strength of granite decreases with increasing temperature^24^.

From the above experimental phenomena, it can be concluded that changes in the micromorphology of granite upon heating to different temperatures and under different cooling modes can be roughly divided into two regimes. When the heating temperature is between 100 and 300 °C, the fracture surface morphology of the specimen generally consists of smooth planes with tiny cracks, and no noticeable change in the crystal structure occurs. Both grain boundaries and transgranular fractures exist. At heating temperatures higher than 300 °C, the original microcracks gradually expand and eventually develop into larger cracks. At the same time, new microcracks are generated. After water cooling, the sample can be seen to have undergone both physical and chemical changes. The structural damage is severe, which in turn leads to changes in the mechanical properties of the rock.

## Discussion

Whether cooled naturally or with water, the degree of damage caused to granite increases with the increase in the heating temperature. The damage that occurs is composed of two main parts. The first is the damage incurred during the heating process, and the other is the damage incurred during the cooling process. The damage that occurs during the cooling process is related to the cooling mode and cooling rate. In this experiment, the heating rate was the same in all cases; thus, the damage caused to the test specimen during the heating process must be the same.

### Changes in the main mineral composition

The XRD equipment selected for this test is the DX2700B diffractometer. The main mineral components of granite when untreated are plagioclase (28%), potash feldspar (19%), quartz (45.6%), mica (6.5%), and other mineral components (8.9%); among these, Plagioclase and quartz account for a large proportion, while mica and potash feldspar account for a relatively small proportion.

**Figure 7:**
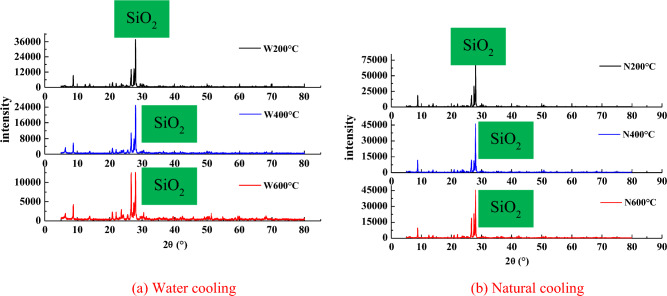
XRD patterns of granite. (**a**) Water cooling, (**b**) Natural cooling.

From the Fig. 7 seen that by comparing the XRD patterns of the granite samples under the two cooling methods, it is found that the diffraction data derived from the four main minerals in granite has not disappeared; however, their diffraction intensity has changed, which indicates that the change in the rock is mainly due a change in the internal structure. From the changes in the diffraction angles corresponding to the maximum diffraction peaks of the four main minerals after heating to different temperatures, it can be seen that the maximum diffraction peaks under the two cooling modes decrease with the increase in temperature. The content of the mineral components will be reduced accordingly. Comparing the two cooling methods, it can be seen that the peak corresponding to the water-cooled sample is lower than that corresponding to the naturally cooled sample, which is mainly due to the physical and chemical effects of the minerals during the cooling process. During the cooling process, due to the capillary action of water, the pores and cracks inside the sample are filled and play a role during cooling. At the same time, water and minerals react chemically, and some minerals will decompose under a certain temperature. (For example, when the temperature is 200 ~ 550 °C, biotite and potassium feldspar will decompose upon getting in contact with water. See Eq. 1 for the specific expression). The naturally cooled sample is placed in the furnace for cooling; however, due to the thermal stress the sample is subjected to for a prolonged period of time, the sample is thermally expanded and is finally cooled to room temperature. The sample cooled by water is subjected to heating during the heating process. After thermal expansion, it is quickly water-cooled, which causes it to shrink rapidly. This results in the sample cracking due to the rapid change in temperature, and the damage caused to the sample mineral particles cannot be recovered.

### Damage mechanism of granite under the two cooling methods

#### (1) Water cooling

The destruction of rock mainly depends on the composition and structure of the rock. Therefore, X-ray fluorescence (XRF) testing was conducted to determine the main elements and mineral composition of granite upon changing the heating temperature; the XRF equipment used in this study is the E3 fluorescence spectrometer produced in Germany. From Tables 1, 2, it can be observed that the main mineral elements have not disappeared, and there are similar changes between the main mineral elements; the only difference is that the content has changed. The results show that the content of SiO_2_ is the highest, with an average value of 67.745%, followed by Al_2_O_3_, with an average content value of 19.347%; furthermore, a small amount of MgO, K_2_O, CaO, Fe_2_O_3_, and P_2_O_5_ are also present. The mineral composition of granite cooled from different temperatures was analyzed via both XRD and XRF. It can be seen that the main minerals in the selected granite are quartz, potash feldspar, plagioclase, and mica, which contain a small amount of other minerals. When the temperature is between 200 and 550 °C, the oxide content is changed, and SiO_2_ increases from 67.457% at 100 °C to 69.022% at 600 °C (an increase of 2.3%); on the other hand, K_2_O decreases from 4.114% at 100 °C to 3.803% at 600 °C (a decrease of 7.6%). This may be because, when the sample is between 20 and 200 °C, the interlayer and adsorbed water will cause hardening of the clay and increase the strength of the rock. When the temperature is higher than 200 °C, thermal cracking can reduce the cohesion of mineral particles and clay, but the mineral particles inside the rock are not significantly affected by thermal stress; therefore, this can not only cause a recoverable elastic deformation (thermal strain expansion) but also no small microcracks are formed. The existing thermal expansion will cause the existing microcracks to close, which leads to an increase in the overall strength of the rock. In addition, by examining the mineral composition of granite, it can be seen that the sample contains a small amount of clay minerals, and the increase in temperature causes the clay to expand and fill or compress the defects inside the rock, which is also a major reason behind the increase in the overall strength of the rock.

**Table 1:** Content of various oxides in granite after heating to different temperatures.

Oxides	100 ℃	200 ℃	300 ℃	400 ℃	500 ℃	600 ℃
MgO	1.51	1.39	1.44	2.14	1.74	1.66
Al_2_O_3_	19.47	19.43	19.04	19.28	19.68	19.18
SiO_2_	67.46	68.18	68.13	66.7	66.98	69.02
K_2_O	4.11	3.76	4.09	3.96	4.19	3.8
CaO	3.61	3.58	3.7	3.91	3.52	3.43
Fe_2_O_3_	2.07	1.91	1.93	2.14	2.15	1.45
MnO_2_	541.7	449.2	488.9	521.2	541.7	332.8
P_2_O_5_	0.59	0.61	0.62	0.62	0.58	0.54

**Table 2:** Content of main elements of granite after heating to different temperatures.

Main element content	100℃	200℃	300℃	400℃	500℃	600℃
Mg	1.24	1.17	1.18	1.75	1.56	1.38
Al	15.81	15.62	15.49	15.66	15.86	15.43
Si	58.91	60.94	59.62	58.03	58.28	62.17
K	8.93	8.03	8.9	8.55	9.11	8.28
Ca	7.3	7.03	7.5	7.86	7.14	6.9
Fe	4.59	4.08	4.28	4.73	4.78	3.15
p	0.61	0.62	0.64	0.63	0.6	0.57
Mn	0.11	0.09	0.1	0.1	0.11	0.07

The mineral composition and element composition inside granite change with the change of temperature (for example, quartz will change phase at high temperature, and biotite will react with water). The oxides in the rock will decompose when they encounter water. However, these reactions will cause the destruction of the mineral crystals, breaking the state of the original rock, which will lead to an increase in the internal defects of granite. As the temperature of the sample increases, the moisture is gradually lost. H_2_O will decompose into free H^+^ ionic bonds through the action of temperature. As H^+^ is in a free state at high temperature, these free H + ionic bonds may react with the minerals of the rock, which will cause pore cracks and result in a complex chemical reaction, such as the dissolution and precipitation of minerals, thereby changing the mineral structure of granite. When the sample is in the mid-temperature stage (300 ~ 400℃), from the SEM micrographs and images, it can be seen that a small number of internal cracks appear in the sample, and these cracks mainly occur between the particles and inside them, indicating the occurrence of thermal cracking. As shown in the figure, due to the effect of temperature, the thermal expansion coefficient changes, causing a change in the rock particles, which gradually change from exhibiting intercrystalline cracks to exhibiting transcrystalline cracks. At this time, the mineral particles exhibit an inconsistent and irreversible thermal expansion. When the thermal stress exceeds the bearing capacity of the surrounding minerals, thermal cracking will occur. The change in temperature gradient is a significant factor that leads to a certain degree of thermal cracking. The higher the temperature, the more severe the thermal cracking.

Granite has an effect on the mineral crystals during the heating–cooling process through endothermic and exothermic reactions. Under the action of high temperature, granite gives rise to thermal cracking or thermal shock. The change in mineral composition changes the physical and mechanical properties of the rock to a certain extent. This is similar to the result reported by Sun^25^. After the heat treatment, the rock will reduce its physical and mechanical properties^26^. When granite is heated, the water inside the rock will evaporate, and it will also evaporate from the pores and cracks of the sample to reduce the pore pressure. This will cause the rock to break and reduce its strength. Heating will also cause thermal stress inside the rock, causing rock thermal damage, which is irreversible. The thermal conductivity of the rock has an important influence on the pores and microcracks inside the rock. When the sample is in a low-temperature stage, the temperature has little effect on the original microcracks inside the rock; therefore, the degree of crack damage of the sample is relatively small. When the sample is in a high-temperature stage and is subjected to a sufficiently large thermal stress so that the pores inside the rock are filled with water, the water will exert a tensile effect on the minerals, increasing the distance between the particles and the interior of the rock. The heat energy will also decrease with the loss of water, which will play a cooling role and also expand the pore cracks inside the rock.

To a certain extent, macrocracks are caused by the accumulation of microdamages. This damage can also be analyzed via an optical microscope. It can be seen that cracks appear on the rock surface, and, as the temperature increases, the cracks become increasingly larger. Even debris appears on the surface. On the one hand, when the rock is heated, the volume expands. As the degree of heating of the rock varies from the surface to the inside, the expansion degree of each part of the rock is different: When the rock is cold, its volume shrinks. On the other hand, since the temperature is different from the surface of the rock to the inside, the degree of shrinkage of each part of the rock is different. After a long time, the different parts of the rock will rise and shrink unevenly, and the internal structure will be destroyed, cracks will occur, and even debris will fall off.

It can also be seen from the SEM micrograph that the number of intragranular cracks in granite is higher at 600 °C than at other temperatures, and it gradually increases with the increase in temperature. Granite is more active under high-temperature conditions, especially when the temperature reaches 600 °C. Indeed, at this temperature, the reaction is the strongest, and the main oxide inside the rock changes, which is quite different from the situation at 100 ~ 500 ℃. This is mainly because, upon encountering water at high temperature, the sample is in, it generates alkaline substances, and the minerals change. Generally, MgO has a lower amount of alkaline substances, while Fe_2_O_3_ has a higher one. For example, from Eqs. 2–6, it can be seen that SiO_2_ in granite reacts with water to form H_4_SiO_4_, etc. It can be observed that the oxides in granite all react with water to form substances containing H + ionic bonds. From the SEM images and microscopic slices, it can be seen that, during the high-temperature stage (500 ~ 600 °C), the internal fracture of the sample is more severe. This may be due to the conversion between the mineral phases, or it may be because some minerals will melt, resulting in some microscopic defects in the rock. The higher the temperature of the sample, the more noticeable the defects inside the rock; as the temperature increases, the number of cracks and the crack width also gradually increase.

After the heat treatment, the sample exhibits not only chemical changes but also physical changes. Under the effect of temperature, due to the difference in particle size and thermal expansion coefficient of various mineral particles in granite, the thermal expansion at the particle boundaries is inconsistent. The tensile and compressive stress (structural thermal stress) generated between the mineral particles or between the particles cause microcracks in granite. Subsequently, the primary and secondary cracks expand and penetrate, causing a deterioration of the macroscopic mechanical properties, and the cracks increase microscopically with the increase in temperature. Additionally, the degree of deterioration of the water-cooled sample is significantly greater than that of the naturally cooled sample. With the increase in temperature, the change in the oxides may be due to the anisotropy between the samples. Although the samples were all taken from the same rock, there may be a certain difference in their mineral content, which may also be one of the reasons due to which the elements in the sample are the same, while their content is different. Finally, the structure of the specimen is destroyed, thereby reducing the strength of the rock.

**Figure 8:**
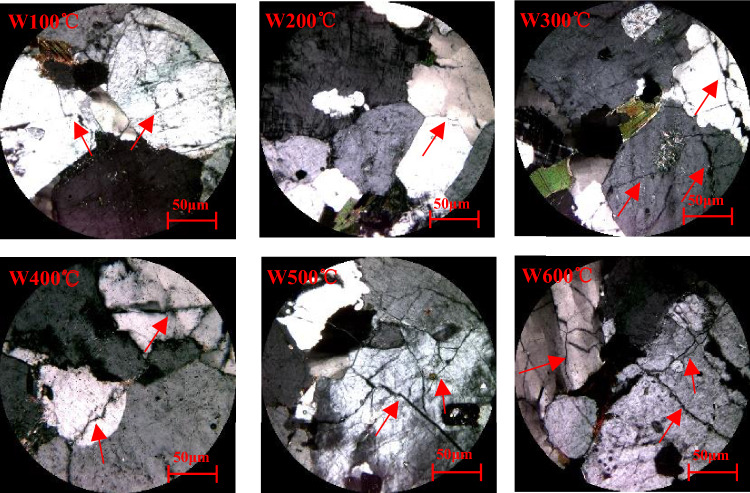
Microscopic slices of granite under water cooling (the red arrow marks the rupture line).

As can be seen from Fig. 8, When the granite temperature reaches 100 and 200 °C and water is encountered, the fast cooling rate will cause a relatively high temperature gradient between the outside and the inside of the specimen, and a relatively high cold shrinkage deformation gradient will also be present. Additionally, as the test specimen is not homogeneous, the cold shrinkage deformation will also be nonuniform, creating tensile stress between the constitutive mineral particles of the test specimen. When the tensile stress exceeds the bond strength, microcracks are generated, causing damage to the test specimen. This damage is mainly due to the cooling of granite. From the microscopic slices of granite under water cooling it can be seen that: (1) As the temperature increases, the degree of damage of the sample gradually increases, which is mainly manifested in the gradual increase in the number and width of cracks; (2) Mineral crystals gradually change from a microcrack state to a multicrack state, and the damage degree of the sample heated to temperatures higher than 400 °C becomes increasingly more severe.

When the heating temperature is higher than 300 °C, a large amount of high-temperature and high-pressure water vapor and air bubbles will be generated for the water-cooled sample^27–30^. When these invade the rock, they will have a “wedging” effect on the test specimen, which will cause the pores in the specimen to expand further and evolve. At the same time, due to chemical reactions of the minerals inside the rock under the action of water, the internal components will be transformed. SiO_2_ and CaO react to form CaSiO_3_, CaO and Fe_2_O react to form ferrite, and CaO and CO_2_ react to form CaCO_3._ In addition, since biotite, which has the chemical formula K(Mg,Fe)_3_AlSi_3_O_10_(OH)_2_, contains (OH)^−^, it tends to thermally decompose from a temperature of 200 °C. When the temperature is higher than 450 °C, biotite begins to thermally decompose, and the thermal decomposition becomes more severe when the temperature reaches 600 °C. When the temperature is between 200 and 550 °C, K-feldspar will degrade into kaolinite or mica due to the phase equilibrium of the K_2_O–Al_2_O_3_–SiO_2_–H_2_O system. Equation 1 is the most relevant chemical equation in this work, while Eqs. 2–6 are other major chemical equations.

1 $$3KAlSi_{3} O_{8} + 12H_{2} O \to KAl_{3} S{\text{i}}_{3} O_{10} (OH)_{2} + 2K^{ + } + 6H_{4} S{\text{i}}O_{4} + 22H^{ + }$$

2 $$SiO_{2} {\text{(quartz)}} + 2H_{2} O \to H_{4} S{\text{i}}O_{4}$$

3 $$K_{2} O + H_{2} O \to 2K^{ + } + 2OH^{ - }$$

4 $$Na_{2} O + H_{2} O \to 2N{\text{a}}^{ + } + 2OH^{ - }$$

5 $$CaO + H_{2} O \to Ca(OH)_{2}$$

6 $$MgO + H_{2} O \to Mg(OH)_{2}$$

In the granite samples cooled in water, SiO_2_ reacts only weakly with water to form H_4_SiO_4_, which has a very low solubility in water. H_4_SiO_4_ fills in the original cracks and defects in granite and thus has a certain restoring effect. CaO and MgO react with water to form Ca(OH)_2_ and Mg(OH)_2_, respectively, which are freed from mineral grains and also have a certain restoring effect on primary cracks and defects. The content of Na_2_O is extremely small, so its reactions have little consequence. Additionally, Zoussi MLS^31^ reported that the quartz crystals inside granite change from the *α* to the *β*phase at 573 °C, causing the volume of quartz grains to increase rapidly, thereby further expanding, widening, and connecting the microcracks inside the specimen. The SEM images in Fig. 5. Figures 3, 4 show that, after the rock has been heated to the same temperature, the degree of damage after water cooling is significantly greater than that after natural cooling; however, the edges of the cracks are relatively blurred after water cooling, some cracks are filled, and dissolution pits are evident. These features result from the hydration of granite.

#### (2) Natural cooling

**Figure 9:**
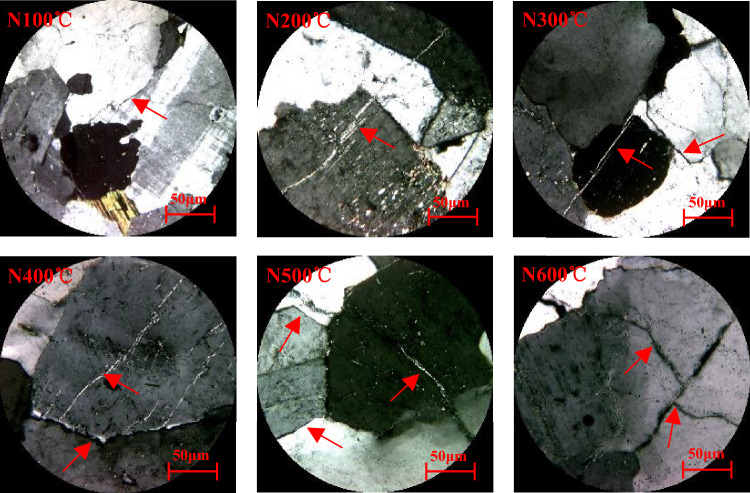
Microscopic slices of the sample under natural cooling (the red arrow marks the rupture line).

As can be seen from Fig. 9, When the temperature is lower than 200 °C, the mineral particles inside the specimens expand during the heating process, and a small number of microcracks are generated due to the uneven thermal expansion deformation, while the primary microcracks remain closed. Small amounts of gas and/or liquid are heated and become volatilized^32^, for example, as water vapor; the loss of this gas and/or liquid improves the contact between the mineral particles, thereby increasing friction. In the natural cooling process, the cooling rate is very slow compared with that under water cooling. The temperature gradient generated inside the specimen is very small. During the natural cooling process, the shrinkage deformation is relatively uniform, and the probability of creating new cracks is very small^33^. From the SEM image in Fig. 6, it can be seen that, after heating the sample to 100 °C, the number of cracks is limited, and they are located mostly along the grain boundaries; thus, when the heating temperature is less than 200 °C, natural cooling can be considered a slow process. Almost no new cracks are generated in the test specimens, but the shapes of the cracks may change, such as when an originally closed crack opens during the shrinking process, or the width of an original crack increases.

When the temperature is greater than or equal to 200 °C, thermal cracking will occur in the test specimen, and the higher the temperature is, the more noticeable the thermal cracking effect will be. This is again caused by chemical effects, but the chemical action during the natural cooling process will cause a very small amount of damage because hydration reactions will be limited. This is the most relevant difference between water cooling and natural cooling. This leads to the damage to naturally cooled granite being less than that in water-cooled granite.

Through the mechanical test, it is found that the granite under the two cooling methods has obvious difference, which is mainly manifested in the compressive strength, and the strength of the water-cooled specimen is significantly lower than that of the naturally cooled specimen. In addition, through microscopic tests, such as scanning electron microscopy and microscopic slice analysis, it can be clearly compared that the cracks of samples after water cooling are more complex than that of samples after natural cooling.

## Conclusions

In this work, the microfracture and failure mode of granites heated to different temperatures and then cooled naturally or with water were analyzed. The main conclusions are as follows:

- (1) For natural cooling, the uniaxial failure mode of granite is mostly splitting and tensile failure, and most failure modes consist of brittle failure after water cooling.
- (2) Analysis of the cross-sectional morphology indicates that the change occurring in granite at different temperatures can be divided into two regimes. When the heating temperature is less than 300°C, there is no clear change in the crystal structure of the sample, and both intergranular and transgranular fractures occur.
- (3) The degree of fracture after cooling with water is greater than that after cooling naturally, as is the degree of transgranular failure. When the heating temperature exceeds 300°C, the original microcracks gradually expand and eventually develop into larger cracks. At the same time, new microcracks are generated.
- (4) After water-cooling, the sample undergoes chemical changes. Severe damage to the crystal structure occurs, and new products are formed, both of which in turn lead to changes in the mechanical properties of the rock.
- (5) From a microscopic point of view, the degradation degree of granite after water cooling is considerably greater than that after natural cooling. This can provide a favorable foundation for heat transfer in artificial heat reservoirs.

## Data Availability

The datasets used and analyzed during the current study are available from the corresponding author on reasonable request.
